# Essential Oils of Lemongrass (*Cymbopogon citratus* Stapf) Induces Apoptosis and Cell Cycle Arrest in A549 Lung Cancer Cells

**DOI:** 10.1155/2020/5924856

**Published:** 2020-01-11

**Authors:** Duong Thu Trang, Thi Kim Van Hoang, Thi Thu Minh Nguyen, Pham Van Cuong, Nguyen Hai Dang, Hong Duyen Dang, Trung Nguyen Quang, Nguyen Tien Dat

**Affiliations:** ^1^Advanced Center for Bio-Organic Chemistry, Institute of Marine Biochemistry (IMBC), Vietnam Academy of Science and Technology (VAST), 18 Hoang Quoc Viet, Caugiay, Hanoi, Vietnam; ^2^Viettri University of Industry, Tien Kien, Lam Thao, Phu Tho, Vietnam; ^3^University of Science and Technology of Hanoi, VAST, 18 Hoang Quoc Viet, Caugiay, Hanoi, Vietnam; ^4^Center for Research and Technology Transfer, VAST, 18 Hoang Quoc Viet, Caugiay, Hanoi, Vietnam

## Abstract

Essential oils were extracted from the culm and leaf of *Cymbopogon citratus* collected from different regions of Vietnam and analyzed using GC/MS. The results showed that citral is the major component accounting for 61.20%–76.46% of the essential oils. The citral content was higher in the essential oil obtained from the leaf than in that from the culm of lemongrass in all regions. In particular, camphene, valerianol, and epi-*α*-muurolol can be used to differentiate essential oils originating from leaves versus culms. The cytotoxic effects of the essential oils on various lung cancer cell lines were evaluated in the present study. All essential oils exhibited cytotoxicity in the tested cells. The Ha Loc leaf essential oil (HLL) exhibited the most potent effects on A549 and H1975 cells, with IC_50_ values of 1.73 ± 0.37 and 4.01 ± 0.30 *μ*g/mL, respectively. The Hy Cuong leaf essential oil (HCL) showed the strongest effect on H1299 cells, with an IC_50_ value of 2.45 ± 0.21 *μ*g/mL. The Kim Duc culm (KDC) essential oil exerted the strongest cytotoxic effects against H1650 cells, with an IC_50_ value of 4.86 ± 0.29 *μ*g/mL. The HLL induced apoptosis and cycle arrest in A549 cells according to flow cytometric analysis and fluorescent nuclear staining assays. The western blot analysis indicated that HLL induced the apoptotic effect by altering the regulating proteins of the apoptosis process such as caspase-3, Bcl-2, and Bax. The data strongly suggested that the intrinsic pathway may play an important role in the apoptotic effects of HLL.

## 1. Introduction

Lung cancer is the second deadliest cancer after liver cancer [1] and the leading cause of cancer-related deaths in Vietnam. In Vietnam, 22,000 people are diagnosed with lung cancer and 19,500 die from the disease yearly. Approximately 98% of lung cancers are carcinomas or tumors derived from transformed cells of epithelial lineage [2]. Recently, nearly four dozen different histopathological variants of lung carcinoma have been recognized. There are two major types of lung cancer accounting for approximately 85% of all lung cancers: small cell lung cancer and non-small-cell lung cancer (NSCLC). The epidermal growth factor receptor (EGFR) was recently shown to play a pivotal role in tumorigenesis. The EGFR-tyrosine kinase inhibitors (EGFR-TKIs) are considered an emerging class of targeted therapeutic agents for the treatment of NSCLCs [3]. Although most EGFR-mutant NSCLCs initially respond to EGFR-TKIs, such as gefitinib (Iressa) and erlotinib (Tarceva), the vast majority of these tumors ultimately become resistant to drug treatment [4]. Currently, EGFR-TKIs significantly extend the progression-free survival of patients with NSCLC.

Natural products provide invaluable opportunities for new drug discoveries due to the unmatched availability of chemical diversity. According to the World Health Organization, more than 80% of the world's population relies on folk medicine for their primary healthcare needs. Medicinal plants have been a useful source of novel anticancer drugs. Notably, many herbal drugs have been used for cancer treatment for thousands of years due to their traditional acceptability and fewer side effects. *Cymbopogon citratus* Stapf. (lemongrass), belonging to the genus *Cymbopogon*, is widely used spices in tropical countries, especially in Southeast Asia. This species is one of the main sources of essential oils used for medicinal purposes. Traditionally, lemongrass has been used for the treatment of gastrointestinal disturbances [5]. In previous studies, the essential oil of *C. citratus* was shown to exert various pharmacological activities, including antimicrobial [6], insecticidal [7], and cytotoxic activities [8, 9]. The main chemical compounds reported in *C. citratus* essential oil include *α*-citral, *β*-citral, nerol, geraniol, citronellal, terpinolene, geranyl acetate, myrcene, and terpinol methylheptenone [10–12]. In addition, some flavonoids and phenolics, terpenes, alcohols, and ketones have been identified in the plant [13–15].

As part of an ongoing search for biologically active natural products from Vietnamese plants, we found that the essential oils of *C. citratus* grown in Phu Tho province exerted potent cytotoxic activities against various cancer cell lines [16]. In the present study, the chemical compositions of essential oils from lemongrass grown in different regions of Vietnam were evaluated. In addition, the cytotoxic effects on different subtypes of NSCLC cell lines and the anticancer mechanism of lemongrass essential oils were investigated preliminarily.

## 2. Materials and Methods

### 2.1. Materials and Essential Oil Preparation

The lemongrasses (*C. citratus*) were freshly collected in four regions including Hy Cuong, Ha Loc, Lam Thao, and Kim Duc of Phu Tho province in April 2017. The samples were taxonomically identified by Dr. Nguyen, The Cuong, Institute of Ecology and Biological Resources (VAST), and voucher specimens were deposited in the Advanced Center for Bio-organic Chemistry. The leaves and culms of each sample (each 500 g) were hydrodistilled in a Clevenger-type apparatus for 4 h, after which the essential oils were separated and dried with anhydrous Na_2_SO_4_. The obtained oils were stored at −5°C until use.

### 2.2. GC/MS Analysis of Essential Oils

Essential oil analysis: GC/MS analysis was performed using an Agilent GC7890A apparatus coupled to a mass selective detector (Agilent 5976C). An HP-5MS fused silica capillary column (60 m × 0.25 mm id. × 0.25 *μ*m film thickness) was used. Helium was the carrier gas with a flow rate of 1.0 mL/min. The inlet temperature was 240°C and the oven temperature program was as follows: 60°C to 220°C at 4°C/min and then at 20°C/min to 240°C with an interphase temperature of 280°C. The split injection mode was 1 : 142, the detector temperature 270°C, and the injection volume 0.1 *μ*L. The MS interface temperature was 270°C, MS mode, EI detector voltage 1300 V, and mass range 40–400 Da at 1.0 scan/s. Identification of components was achieved based on their retention indices and by comparison of their mass spectral fragmentation patterns with those stored on the MS library (NIST 08, Wiley 09). Component relative contents were calculated based on the total ion current without standardization. MassFinder 4.0 software was used for data analysis.

### 2.3. Cell Lines and Cell Culture

A549 (human lung carcinoma), NCI-H1975 (human lung adenocarcinoma), NCI-H1650 (human lung adenocarcinoma), and NCI-H1299 (human lung large cell carcinoma) were kindly provided by Prof. Jeong-Hyung Lee, Department of Biochemistry, College of Natural Sciences, Kangwon National University, Korea. The cells were cultured at 37 C in RMPI1640 medium supplemented with 10% fetal bovine serum (FBS), 100 U/mL penicillin, and 100 *μ*g/mL streptomycin in a 5% CO_2_ incubator.

### 2.4. Cytotoxicity Assay

The viability of cells was evaluated using the 3-(4,5-dimethylthiazol-2-yl)-2,5-diphenyl tetrazolium bromide (MTT) method. The cells were seeded in 96-well plates at a concentration of 1 × 10^5^ cells/well and treated with various concentrations of essential oils (0.3 1, 3, 10, and 30 *μ*g/mL) and incubated in a humidified 5% CO_2_ atmosphere at 37°C. After 48 h incubation, 0.5 mg/mL MTT was added to each well and incubated for another 4 h. After removing the supernatant, formazan crystals were dissolved in isopropanol and the OD values were measured at 570 nm using a microplate reader. Camptothecin was used as a positive control.

### 2.5. Flow Cytometry Analysis for Cell Cycle

A549 cells were plated in 6 cm plates and allowed to attach for 24 h and then treated with or without essential oils for 48 h. Only adherent cells were harvested and then washed twice with ice-cold PBS. After fixed gently in ice-cold 70% EtOH and incubated for 2 h at −4°C, cells were washed with ice-cold PBS twice. Then, fixed cells were incubated with 1 mg/mL propidium iodide (PI) (Invitrogen, USA) in PBS at room temperature for 30 min. Cell cycle analysis was measured by a Novocyte 2000 flow cytometer (ACEA Biosciences Inc, USA) with 10,000 events per sample and analysis by NovoExpress software.

### 2.6. Flow Cytometry Analysis for Apoptosis

Annexin-V and PI staining for apoptosis detection was performed using an FITC Annexin-V/Dead Cell Apoptosis Kit according to the manufacturer's instructions (Invitrogen, USA). Briefly, cells were treated with various concentrations of essential oils and incubated for 48 h. The cells were then collected by trypsinization, washed 2 times with cold PBS, suspended in 100 *μ*L of a binding buffer (diluted from 10x binding buffer), and stained with 5 *μ*L PI (50 *μ*g/mL stock solution) and 5 *μ*L FITC-labeled Annexin-V in the dark for 15 min at room temperature. The cells were analyzed by Novocyte 2000 (ACEA Biosciences Inc, USA). The percentages of Annexin-V + /PI− (apoptosis cells), Annexin-V/PI− (living cells), and Annexin-V + /PI+ (necrotic cells) staining were determined after marking for the positive and negative population.

### 2.7. Hoechst 33342 Nuclear Staining Assay

Briefly, A549 cells were seeded in 6-well plates at a density of 2.5 × 105 cells/mL for 24 h and then treated with or without essential oils. After 48 h, the culture medium was removed and the cells were washed twice with PBS. The cells were stained with Hoechst 33342 solution (2′-[4-ethoxyphenyl]-5-[4-methyl-1-piperazinyl]-2,5′-bi-1H-benzimidazole trihydrochloride trihydrate) at a concentration of 5 *μ*g/mL at 37°C for 30 min in the dark. The stained cells were observed using a fluorescence microscope (CKX53, Olympus, Tokyo, Japan) for determining any nuclear structural changes and apoptotic bodies.

### 2.8. Western Blot Analysis

A549 cells were seeded in a 6-well plate at a density of 2.5 × 10^5^ cells/mL for 24 h and then treated with or without essential oils. After 48 h, the cells were harvested and lysed with ice-cold lysis buffer containing 150 mM NaCl, 50 mM Tris-HCl (pH 7.4), 1 mM EDTA, 1% NP-40, 5 mM sodium orthovanadate, and protease inhibitor cocktail (BD Biosciences, USA) to extract the total protein. Protein lysates were centrifuged at 22,000 ×g for 10 min at 4°C. Protein concentration in the supernatants was determined using the Bradford method. A total of 20 *μ*g proteins were separated by 12% SDS-PAGE and then transferred to a PVDF membrane (Thermo Fisher Scientific, Inc). The membrane was blocked with 5% nonfat skim milk at room temperature for 1 h, followed by washing with PBS containing 0.1% Tween-20. Thereafter, the membranes were probed with the indicated primary antibodies (Bax, Bcl-2, and caspase-3, 1 : 1,000 dilution) overnight at 4°C. Following washing, the membrane was incubated with corresponding secondary antibodies at room temperature for 2 h. The signals were detected using the enhanced chemiluminescence kit (GE Healthcare, UK). *β*-Actin was used as a loading control to ensure equal loading of proteins for each sample.

### 2.9. Statistical Analysis

Results are given as the mean standard error of the mean (SEM). Statistical analyses were performed with GraphPad Prism 6.0 (GraphPad Software, San Diego, CA).

## 3. Results and Discussion

### 3.1. Identification of Components of Essential Oils by GC/MS Analysis

The essential oils from the culms and leaves of *C. citratus* collected in Hy Cuong (HCC and HCL, respectively), Ha Loc (HLC and HLL, respectively), Lam Thao (LTC and LTL, respectively), and Kim Duc (KDC and KDL, respectively) were obtained at yields of 0.53%–0.71% (Table 1). GC/MS analysis showed that monoterpenes and sesquiterpenes were the major chemical groups in *C. citratus* essential oils. However, the contents of the eight hydrodistilled oils varied greatly. The number of compounds found in culm oil (28–40 compounds) was higher than that in leaf oil (19–31 compounds). Notably, camphene, valerianol, and epi-*α*-muurolol were found only in culm oil but not in leaf oil. The myrcene content was higher in leaf oil than in culm oil. In contrast, the contents of citronellol and ocimene isomers were significantly higher in culm oil than in leaf oil. Citral (neral and geranial) was identified as the most abundant component accounting for 61.20%–76.46% of both essential oils. Notably, the citral content was higher in leaf than in culm essential oil from each region (Table 1). This composition was in the same range reported in previous studies [17]. Although the composition of *C. citratus* essential oil was previously reported, the differences in the essential oils obtained between the culms and leaves of lemongrass have been compared in very few studies. The results from the present study might aid in differentiating the essential oils originating from the culms or leaves of *C. citratus*.

### 3.2. Cytotoxic Effects of Essential Oils of *C. citratus* against Various Lung Cancer Cell Lines

To evaluate the cytotoxic activities of the *C. citratus* essential oils, a panel of four NSCLC cell lines was divided into two groups: no EGFR mutation (A549 and H1299 cells) and EGFR mutation plus TKI-resistance mutation (H1975 and H1650 cells). The cellular proliferation of this panel of four NSCLC cell lines was examined by MTT assay over a 48 h period in the presence of increasing concentrations of various *C. citratus* essential oils. Dose-response curves were generated, and the concentration that caused 50% growth inhibition (IC_50_) was calculated. Consequently, both *C. citratus* oils showed cytotoxic effects. HLL exhibited the most potent effects on the A549 and H1975 cells, with IC_50_ values of 1.73 and 4.01 *μ*g/mL, respectively. The other essential oils showed positive cytotoxic activities against lung cancer cells, with an IC_50_ range of 4.25–8.93 *μ*g/mL (Table 2). Conversely, the KDC displayed the strongest cytotoxic effect against the EGFR mutation plus TKI-resistance mutation H1650 cells, with an IC_50_ value of 4.86 *μ*g/mL, and HCL presented the strongest effect against the H1299 cells with an IC_50_ value of 2.45 *μ*g/mL (Table 2). Among the tested oils, HLL exhibited to be the most sensitive one to A549 cells compared to other samples. Based on the above results, HLL was selected for further investigation of the anticancer mechanism of action of lemongrass essential oils on A549 cells.

### 3.3. Investigation on the Mechanism of Action of Lemongrass Essential Oils in A549 Cells

**Figure 1:**
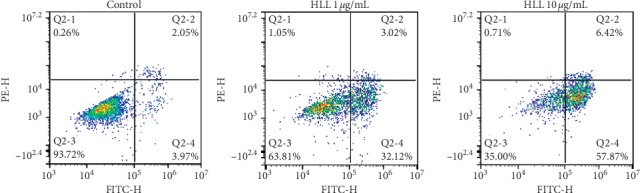
Apoptotic effect of HLL at different concentrations (0, 1, and 10 *μ*g/mL) in A549 cells analyzed with FITC Annexin-V/Dead Cell Apoptosis Kit with FICT Annexin-V and PI, using flow cytometry after 48 hours of treatment. Q2-1 necrosis, Q2-2 late apoptosis, Q2-4 early apoptosis, and Q2-3 viable cell.

**Figure 2:**
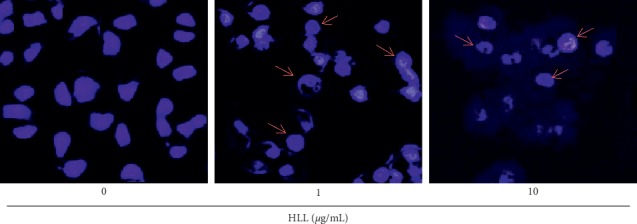
Cell apoptosis observed by Hoechst 33342 staining. A549 cells treated with HLL at concentrations 0, 1, and 10 *μ*g/mL for 48 h (magnification, 400x). Arrows show the dead cells with apoptotic bodies.

Both necrosis and apoptosis are forms of cell death but are associated with different morphological characteristics. FACS analysis using Annexin-V-FITC and propidium iodide staining was used to assess the effect of HLL on apoptosis in A549 cells. Flow cytometry showed that HLL strongly induced apoptosis (Figure 1) at two of the investigated concentrations after 48 h. Apoptosis was induced in 35.14% of A549 cells (32.12% in early apoptosis and 3.02% in late apoptosis) after 1 *μ*g/mL HLL treatment. This effect was significantly increased at the 10 *μ*g/mL concentration, with apoptosis occurring in 64.29% of cells (57.87% early apoptosis and 6.42% late apoptosis). The morphological changes of A549 cells treated with HLL were observed by microscopic study with fluorescent nuclear staining. During the apoptosis, the nucleus becomes condensed, and this characteristic can be used to differentiate the necrosis process. Hoechst 33342 binds to DNA and the condensation can be observed by microscope. As shown in Figure 2, compared to untreated cells, typical morphological alterations including cell shrinkage, chromatin condensation, and nuclear fragmentation were observed in HLL treatment groups. Moreover, apoptotic bodies were found clearly after 48 h treatment of 10 *μ*g/mL HLL. Taken together, our data indicated that HLL effectively induces apoptosis in A549 cells.

**Figure 3:**
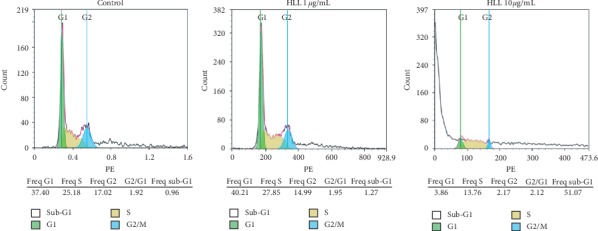
HLL dose-dependently induced cell cycle arrest at sub-G1 phase in A549 cancer cells was analyzed using flow cytometry. The DNA histogram shows the distribution and the percentage of cells in phases of the cell cycle.

The cell cycle is closely associated with apoptosis, and cell cycle arrest leads to apoptosis via effects on various signaling molecules and regulatory proteins. Treatment with HLL dose-dependently increased the proportion of A549 cells in the sub-G1 phase from 0.96% to 1.27%, and 51.07% after treatment with 0, 1, and 10 *μ*g/mL HLL, respectively (Figure 3). Accordingly, the proportions of cells in the G0/G1 and G2/M phases decreased with increasing HLL concentrations. Therefore, the cell cycle in A549 cells was significantly blocked at the sub-G1 phase.

**Figure 4:**
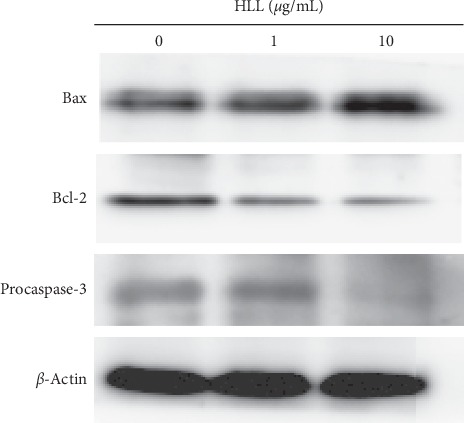
Effects of HLL on the expression levels of Bax, Bcl-2, and procaspase-3 in A549 cells. A549 cells were treated with different concentrations (0, 1, and 10 *μ*g/mL) of HLL for 48 h. Proteins were extracted, and then Bax, Bcl-2, procaspase-3, and *β*-actin expressions were analyzed by western blot analysis.

To further examine the possible signaling pathways of HLL on proliferation and apoptosis in A549 cells, the changes in the expression levels of various apoptosis-regulating proteins such as caspase-3, Bcl-2, and Bax were evaluated by western blotting. HLL-treated A549 cells demonstrated a significant decrease in the expression level of Bcl-2 and a marked increase in the Bax level. As depicted in Figure 4, the exposure of A549 cells to HLL resulted in the downregulation of procaspase-3, indicating the cleavage of caspase-3, and in turn denoted the activation of caspase-3. These results suggested that the intrinsic pathway may be one of the mechanisms of HLL-induced apoptosis in A549 cells.

Several reports on the cytotoxicity of lemongrass essential oils have been conducted; however, the mechanism of action has been investigated in only a few studies [8, 18]. An ethanol extract of lemongrass exhibited significant anticancer properties in lymphoma and leukemia models and effectively induced apoptosis in a recent study [19]. A proapoptosis effect of the polysaccharide fraction of *C. citratus* has also been demonstrated [20]. Citral (neral and geranial), the major component of *C. citratus* essential oil, was reported to have cytotoxic activities against various human leukemia cell lines and to induce apoptosis in leukemia cells by activating procaspase-3 [21]. In the present study, the citral content was higher in leaf than in culm essential oils. Myrcene was the second abundant component in the *C. citratus* essential oils. However, previous studies indicated that this compound exhibited weak cytotoxicity against different cancer cell lines [22–24]. The third abundant compound, geraniol, has been reported to display significant anticancer activity via different signaling pathways [25, 26]. Based on this observation, citral and geraniol may contribute to the cytotoxic activities of *C. citratus* essential oils.

## 4. Conclusion

The essential oils of *C. citratus* from different regions of Vietnam were analyzed using GC/MS. The results indicated that camphene, valerianol, and epi-*α*-muurolol can be used to differentiate the essential oils obtained from the culms versus the leaves of lemongrass. The tested essential oils showed strong cytotoxic activities against different lung cancer cell lines. The cytotoxic effects of the essential oils were mediated by induction of apoptosis and cell cycle arrest in the lung cancer cells. The findings from the present study suggest that the essential oils of lemongrass have potential in cancer prevention.

## Figures and Tables

**Table 1:** Chemical composition and yield of *C. citratus* essential oils collected in Phu Tho province.

Components	(RI)	Hy Cuong		Ha Loc		Lam Thao		Kim Duc	
		HCL (%)	HCC (%)	HLL (%)	HLC (%)	LTL (%)	LTC (%)	KDL (%)	KDC (%)
*α*-pinene	940	—	0.27	—	0.13	—	—	—	—
Camphene	956	—	0.58	—	0.30	—	0.23	—	0.23
6-methylhept-5-en-2-one	988	0.96	0.72	1.23	0.71	0.93	0.79	0.82	0.58
Myrcene	993	8.61	5.44	10.30	3.82	9.38	4.76	8.87	3.09
Dehydro-1,8-cineole	997	—	—	—	0.14	—	—	—	—
Limonene	1035	—	—	2.19	0.30	—	0.11	—	—
(*Z*)-*β*-ocimene	1039	1.07	3.80	0.84	3.12	1.23	3.26	0.92	2.52
(*E*)-*β*-ocimene	1050	0.56	1.72	0.51	1.40	0.61	1.50	0.51	1.19
*γ*-Terpinene	1064	—	—	0.17	—	—	—	—	—
Linalool	1104	1.26	1.42	1.33	1.40	1.23	1.47	1.03	1.22
Lavandulol	1148	0.18	—	0.23	0.10	0.41	0.34	0.35	0.25
Citronellal	1156	0.26	0.56	0.20	0.31	0.13	0.59	0.22	0.47
*trans*-chrysanthemol	1158	0.19	—	0.25	—	—	—	0.14	—
Isoneral	1164	0.77	0.67	1.10	0.74	1.29	1.28	1.33	1.02
*p*-Mentha-1,5-dien-8-ol	1172	—	0.19	0.17	0.59	—	—	2.10	—
Borneol	1179	—	0.25	—	—	—	0.16	—	0.25
Isogeranial	1182	1.10	1.04	1.56	1.26	1.98	1.96	—	1.74
*α*-terpineol	1201	—	0.20	—	0.22	—	0.17	0.20	0.23
Citronellol	1232	0.74	1.81	0.48	1.54	0.70	1.37	0.57	1.91
Nerol	1234	0.36	0.32	0.35	0.58	0.24	0.24	0.29	0.73
Neral	1245	33.25	26.72	32.68	27.51	32.12	30.16	31.02	27.52
Geraniol	1254	3.79	3.10	4.33	3.51	3.76	3.36	3.69	3.49
Geranial	1274	42.21	34.87	40.19	34.98	40.58	38.97	38.32	34.68
2-undecanone	1296	0.14	—	—	—	—	—	0.12	—
Dimethoxy-(*Z*)-citral	1321	—	—	—	—	—	—	1.49	0.55
Methyl geranate	1323	—	—	—	0.14	—	0.12	—	0.16
Dimethoxy-(*E*)*-*citral	1344	—	—	—	—	—	—	3.09	1.17
Geranic acid	1353	—	—	—	0.19	—	—	—	—
Eugenol	1369	—	—	—	—	—	—	0.48	0.30
Geranyl acetate	1385	0.52	0.31	1.09	0.25	1.02	0.51	1.10	0.66
*α*-Cedrene	1433	—	—	—	—	—	—	0.24	0.33
(*E*)-Caryophyllene	1438	0.32	0.60	0.20	0.47	0.33	0.26	0.40	0.56
*α*-*trans*-bergamotene	1446	0.22	0.41	—	0.27	—	0.13	0.14	0.23
*α*-humulene	1473	—	—	—	0.14	—	—	—	—
Germacrene D	1499	—	0.23	—	0.27	—	—	0.34	0.44
*β*-chamigrene	1500	—	—	—	0.15	—	—	—	—
*δ*-Selinene	1506	—	0.31	—	0.30	—	—	—	0.13
*α*-Muurolene	1514	—	—	—	0.27	—	—	—	—
*cis*-dihydroagarofuran	1528	—	0.32	—	0.30	—	—	—	0.24
*γ*-cadinene	1531	—	0.27	—	0.17	—	—	—	—
*δ*-cadinene	1538	—	0.69	—	0.68	—	0.37	0.12	0.75
(*E*)-*γ*-bisabolene	1543	—	0.39	—	0.24	—	—	—	0.19
Caryophyllene oxide	1606	0.32	—	—	0.18	0.22	—	0.18	—
5-Epi-7-epi-*α*-eudesmol	1626	0.20	0.78	—	0.60	—	0.34	0.13	0.85
Cedrol	1631	—	—	—	-	—	—	0.29	0.29
Valerianol	1657	—	0.48	—	0.35	—	0.22	—	0.58
Epi-*α*-muurolol	1663	—	0.87	—	0.76	—	0.41	—	0.89
Hinesol	1666	—	0.33	—	—	—	—	—	0.38
Epi-*α*-cadinol	1666	—	—	—	0.26	—	—	—	—
*α*-cadinol	1676	0.42	1.56	—	1.48	0.48	0.88	0.34	1.92
Neo-intermedeol	1685	—	1.07	—	—	0.37	0.69	—	—
Intermedeol	1687	—	—	—	—	—	—	0.13	—
(*Z*, *Z*)-Farnesol	1727	—	—	—	—	—	—	—	0.14
(*E*, *E*)-Farnesol	1732	—	—	—	—	—	—	—	0.14
(*E*, *Z*)-Farnesol	1752	—	—	—	—	—	—	—	0.17

Total identified (%)		97.60	92.30	99.40	90.13	97.01	94.65	98.97	92.37
Yield (%)		0.63	0.57	0.68	0.53	0.71	0.56	0.61	0.53

^a^Yield calculated based on the fresh materials; RI: retention index; HCC: culm oil collected in Hy Cuong; HCL: leaf oil collected in Hy Cuong; HLC: culm oil collected in Ha Loc; HLL: leaf oil collected in Ha Loc; LTC: culm oil collected in Lam Thao; LTL: leaf oil collected in Lam Thao; KDC: culm oil collected in Kim Duc; KDL: leaf oil collected in Kim Duc.

**Table 2:** The IC_50_ values of *C. citratus* essential oils on various lung cancer cell lines.

Essential oils^a^ (*μ*g/ml)	Wild type of EGFR and TKI-resistance		Mutation of EGFR and TKI-resistance	
	A549	H1299	H1650	H1975
HCL	3.84 ± 0.43	2.45 ± 0.21	6.24 ± 0.52	5.36 ± 0.27
HCC	3.77 ± 0.28	3.56 ± 0.40	6.81 ± 0.75	6.16 ± 0.54
HLL	1.73 ± 0.37	3.37 ± 0.18	7.89 ± 0.64	4.01 ± 0.30
HLC	4.50 ± 0.30	4.94 ± 0.47	8.71 ± 0.36	4.25 ± 0.53
LTL	4.98 ± 0.68	5.79 ± 0.27	6.52 ± 0.29	4.74 ± 0.07
LTC	4.40 ± 0.75	6.56 ± 0.39	8.36 ± 0.08	5.83 ± 0.25
KDL	4.25 ± 0.32	7.50 ± 0.93	6.15 ± 0.28	5.46 ± 0.93
KDC	5.28 ± 0.59	8.93 ± 0.50	4.86 ± 0.29	8.49 ± 0.68
Camptothecin^b^	0.48 ± 0.02	0.49 ± 0.05	0.42 ± 0.01	0.42 ± 0.05

^a^Experiments were carried out in triplicate; ^b^positive control.

## Data Availability

The data used to support the findings of this study are included within the article.
